# SAVE: Spectrum-Aided Visual Enhancement for AI-Based Skin Cancer Detection

**DOI:** 10.3390/diagnostics16121864

**Published:** 2026-06-16

**Authors:** Hung-Yi Huang, Yaswanth Nagisetti, Arvind Mukundan, Riya Karmarkar, Sahaya Ashik Libu, Tao-Yuan Liu, Hsiang-Chen Wang

**Affiliations:** 1 Department of Dermatology, Ditmanson Medical Foundation Chiayi Christian Hospital, Chiayi 60002, Taiwan; huanghungyi1@gmail.com; 2 Department of Mechanical Engineering, National Chung Cheng University, 168, University Rd., Min Hsiung, Chiayi 62102, Taiwan; yaswanthnagisatti@gmail.com; 3 Department of Biomedical Imaging, Chennai Institute of Technology, Sarathy Nagar, Chennai 600069, Tamil Nadu, India; arvindmukund96@gmail.com; 4 Department of Computer Science Engineering, School of Engineering and Technology, Sanjivani University, Kopargaon 423603, Maharashtra, India; 5 Department of Integrated Bachelor of Technology, School of Engineering and Technology, Sanjivani University, Kopargaon 423603, Maharashtra, India; karmakarriya345@gmail.com; 6 Department of Computer Science Engineering, Jeppiaar Engineering College, SH 49A, Chennai 600119, Tamil Nadu, India; ashiklibu11@gmail.com; 7 Department of Pediatrics, Kaohsiung Armed Forces General Hospital, 2, Zhongzheng 1st Rd., Lingya District, Kaohsiung City 80284, Taiwan; 8 Department of Medicine, National Defense Medical University, No. 161, Sec. 6, Minquan E. Rd., Neihu District, Taipei City 11490, Taiwan; 9 Department of Medical Research, Dalin Tzu Chi General Hospital, No. 2, Min-Sheng Rd., Dalin Town, Chiayi 62247, Taiwan; 10 Hitspectra Intelligent Technology Co., Ltd., Kaohsiung City 80661, Taiwan

**Keywords:** medical image reconstruction, novel signal processing, supervised learning, hyperspectral imaging, YOLO, SSD, Skin cancer detection, clinical workflows

## Abstract

**Background/Objectives:** The early identification of skin cancer by standard RGB dermoscopy is a clinical difficulty because of the complex visual differences between impacted lesions and healthy tissue. **Methods:** For the biomedical challenge, a novel approach to signal processing and image reconstruction is introduced in this study, called the spectrum-aided visual enhancer (SAVE). The proposed SAVE mechanism aims at reconstructing the diagnostically relevant spectral information from the conventional RGB dermoscopic images using the principles of hyperspectral imaging (HSI) and band selection (BS). After quality control and pre-processing, the images in the ISIC2019 dataset were selected, with 865 images that contain basal cell carcinoma (BCC), seborrheic keratosis (SK), and actinic keratosis (AK) lesions. To reduce data leakage, the dataset was split into training, validation, and testing subsets of 70%, 20%, and 10%, respectively. Five supervised deep learning object detection models were trained and tested on the conventional RGB image dataset and on the SAVE-enhanced dataset. Five supervised deep learning object detection models, namely, YOLOv8, YOLOv10, YOLOv11, SSDLite, and SSD, were trained and tested on the conventional RGB image dataset and the SAVE-enhanced dataset. Additional repeated experimental assessments and statistical comparisons were also carried out to evaluate the improvement in performance. **Results:** The experimental results showed that the SAVE-based pre-processing always yielded better performance in terms of lesion detection than conventional RGB image processing. The SAVE framework for SSD was evaluated and compared with all other evaluated models and was found to be the most successful, with an accuracy of 96%, a precision of 97%, a recall of 96%, and an F1 score of 96%. **Conclusions:** The results indicate that the proposed SAVE framework could be a promising RGB-compatible spectral enhancement technique for boosting skin cancer detection and computer-aided dermatologic analysis with the aid of AI.

## 1. Introduction

The latest statistics from the American Cancer Society indicate that in 2026, approximately 112,000 adults in the USA are expected to be diagnosed with invasive melanoma [1]. Skin cancer treatment remains a crucial issue in modern healthcare, and this type of cancer is significantly more likely to affect people with white skin [2]. The incidence of skin cancer is strongly correlated with age, showing a notable increase after 50 years and peaking in individuals aged 75 and above [3]. Melanoma and non-melanoma skin cancer (NMSC) are the two main categories of skin cancer [4]. If left undetected, NMSC rarely penetrates the deeper epidermal layers but can be easily removed when caught early [5]. NMSC is found more frequently than any other cancer combined and is predicted to occur in more than 400,000 cases per year in the UK by 2025 [6]. NMSC is mainly differentiated into two types known as basal cell carcinoma (BCC) and squamous cell carcinoma (SCC) [7].

Most NMSCs are BCC and occur mostly in the head and neck regions [8]. BCC accounts for 50% of all cancers in the United States. Although the mortality from this cancer is very low [9]. Actinic keratoses (AKs), which are flat, hyperkeratotic, scaly skin lesions, may progress to cutaneous squamous cell carcinoma; however, the risk ranges from 0.025% to 16% [10]. AKs frequently appear on the sun-exposed skin of Caucasian adults, manifesting as rough, dry, scaly, or crusted lesions that can be skin-colored or tanned, often with a reddened base [11]. Seborrheic keratoses (SKs) are the most common epithelial tumors in clinical and telemedicine environments. These largely benign lesions are frequently biopsied and removed for cosmetic purposes [12]. Most of the time, SKs are confused with SCC and other skin cancers due to their visual similarity to other skin cancers [13]. SKs can present as papules, macules, or plaques, with colors ranging from tan to black, and they are noted for their distinct verrucous, ‘stuck-on’ appearance. Additionally, they may show exophytic or hyperplastic growth features [14]. Artificial intelligence (AI) has now become a revolutionary tool in many sectors, including the diagnostic pathway. It has especially received much attention in dermatology applications, thus improving the automation of skin disorder diagnosis and treatment [15].

Skin cancer remains one of the most prevalent and rapidly increasing forms of cancer worldwide, creating a significant global healthcare burden and emphasizing the importance of early diagnosis and timely clinical intervention [16,17]. There have been recent epidemiological reports of significant rises in the incidence of melanoma and non-melanoma skin cancer in a wide variety of populations, especially in elderly people and in areas where levels of ultraviolet radiation are high [18]. At the same time, the development of computer-aided dermatologic diagnostic systems has progressed, thanks to the development of artificial intelligence and deep learning, and they have been able to contribute to the accurate detection of lesions and the process of clinical decision-making [19]. In the last few years, hyperspectral imaging (HSI) and spectral-enhancement methods have shown great promise in enhancing the visualization and characterization of lesions and tissues beyond that of standard RGB images. Despite these developments, however, there are still challenges such as high equipment costs, high computational load, and limited clinical accessibility that hinder the use of hyperspectral imaging systems in the general dermatology setting. Considering these limitations and the rising demand for easily implementable RGB-compatible spectral enhancement methods, the proposed spectrum-aided visual enhancer (SAVE) framework aims to enhance the visualization of skin lesions and assist skin cancer detection using reconstruction directly from conventional RGB dermoscopic images through the use of hyperspectral-inspired reconstruction techniques.

Computer vision, in particular, has adopted machine learning to revolutionize computer-aided diagnosis and prediction models for skin cancer [20]. In 2021, in a study by Pradhumn et al. [21], a mobilenet-based model was used and trained on roughly 39,000 dermoscopic images of skin lesions taken from HAM10000. For seven categories of skin lesions, the overall performance of the model was validated to provide 89% of weighted average precision, 73% of recall, and 76% of F1 score. Guan-Yi et al.’s [22] work in 2023 has proven the performance of lightweight YOLOv7 in detecting AK lesions with a precision of 93%, a recall of 80%, and an mAP of 87% with the training of 400 AK images. Ahalya et al. [23] proposed a convolutional neural network (CNN) model in 2024 that had 90% sensitivity, 94% specificity, and 95% accuracy when tested on the dataset collected from Kaggle. Additionally, the segmentation results are visualized to confirm the model’s efficacy. In 2022, Rabbia et al. [24] introduced a new model for skin cancer detection with feature fusion. The model performed remarkably well on 1000 images from the dermis dataset and achieved 99.4% accuracy, 98.7% precision, and 98.66% recall. However, these methods yield promising results for skin cancer detection by exploiting only a small amount of image data in the three color channel values. Moreover, if advanced technologies such as hyperspectral imaging (HSI) and band selection (BS) can be integrated, then the performance could be greatly improved.

Using the hyperspectral imaging technique allows us to take advantage of a bandwidth of 10 nm or less, allowing for the detection of fine-scale spectral features that would otherwise be lost [25]. This technique acquires hundreds of images for a single spatial area at different wavelengths [26], which is substantially more spectral data than multispectral imaging and RGB imaging [27]. HSI produces a 3D dataset, termed a ‘hypercube’, that combines 1D spectral and 2D structural information [28]. Hyperspectral imaging, as described, merges imaging with spectroscopy and provides spectral signatures alongside spatial data for clear morphological visualization. It is shown to be valuable for diagnosing, monitoring microenvironments, and assessing solid tumor margins [29]. Some recent applications of HSI are the analysis of meat quality and safety [30], identifying the different types of farmland soil microplastics [31], classification of large-scale agricultural crops [32], cancer detection [33], etc. Due to the amount of information produced by HSI, the subsequent data processing must be made as quickly as possible, and the use of conventional image processing methods is not preferable [34].

BS is used to select a small subset of hyperspectral bands; it is a common technique designed to minimize computational complexity and remove great redundancies in the spectrum while retaining all useful spectral information of objects [35]. Research has identified six primary hyperspectral BS approaches, namely, ranking-based, search-based, clustering-based, sparsity-based, embedding learning-based, and hybrid scheme-based [36]. In 2023, Nan Weng et al. [37] presented a new hyperspectral anomaly detection (HAD) algorithm named the multi-prior graph autoencoder (MPGAE), including band selection, adaptive weight strategy, and graph regularization. The BS component assists in reducing such unwanted spectral information, enhances discriminant power, and lowers computation needs. In the year 2021, Xudong et al. [38] proposed a novel technique of band selection specifically for target detection, which is called constrained-target band selection with subspace partition (CTSPBS) for hyperspectral target detection, and this mechanism is used for detecting marine benthos in mariculture.

The study describes a mechanism called a spectrum-aided visual enhancer (SAVE); it computes spectral information from a given RGB image and applies BS to select bands that have the most effective spectral signal for enhancing the visuals of different skin conditions. Various object detection models were trained using RGB and SAVE datasets to determine the efficiency of the SAVE mechanism in diagnosing AK, BCC, and SK skin cancers. Yolov8, Yolov10, Yolov11, SSDLite, and SSD models are trained on both the RGB dataset and the SAVE dataset. Then, the qualities of the obtained results from SAVE-based models are compared to those of the conventional RGB-based models.

## 2. Methods

### 2.1. Dataset

Specifically, this research used a dataset comprising 25,331 dermoscopic images of skin lesions from the ISIC Challenge 2019 training dataset. From this set, 867 AK images, 3323 BCC images, and 1316 SK images were selected. After excluding, through sorting capabilities, images of low quality or poor definition, with hair or revealing high cancer spread, the final dataset set contained 257 AK images, 286 BCC images, and 322 SK images; 865 in total. Annotation was done using Roboflow, where each image was cast into a singular class, with each image labeled according to its cancer lesion type: either AK, BCC, or SK. The annotated images were then divided into three sets systematically: training, 70%; validation, 20%; and test, 10%. The training set was augmented by flipping over vertically and horizontally, which tripled the size of the training set. No more augmentation strategies were used since the above two transformations reflect realistic scenarios, and if other techniques are used, then the real properties of cancer lesions are changed. To standardize the size of the images, all were resized or cropped to the size 640 × 640 pixels. Up to this stage, all processes remain the same for both datasets. All the images were then passed through the SAVE mechanism to obtain the SAVE dataset.

The performance of the deep learning models is evaluated based on precision, recall, F1 score, and overall accuracy for both RGB and SAVE datasets. The same parameters were used for both datasets, and all models were trained for 500 epochs with a Kaggle P100 GPU. While performing the training and testing, the confidence threshold was fixed at 60%, and the batch size was set to 32. All the other parameters were left at their typical levels, and all the evaluation metrics were obtained strictly from the test set.

The ISIC2019 database comprised 25,331 dermoscopic images of various skin lesion types. Images were selected for this study only for actinic keratosis (AK), basal cell carcinoma (BCC), and seborrheic keratosis (SK), as these lesion types often have fine visual features that are difficult to differentiate with traditional RGB dermoscopy.

In total, 867 AK images, 3323 BCC images, and 1316 SK images were extracted from the images. A quality control (QC) filtering was then applied to increase the quality of annotation and the visibility of lesions. Photos were not included if there were severe light reflections, significant hair occlusion, unclear margins of the lesion, poor focus, low contrast, or if the lesion was spread out too much and could not be localized.

**Figure 1:**
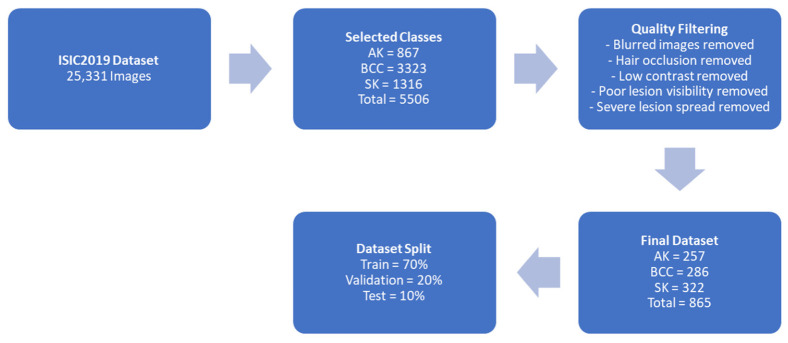
Dataset reduction flowchart.

Following the filtering, 257 AK images, 286 BCC images, and 322 SK images were left in the final dataset, for a total of 865 dermoscopic images presented in Table 1 and Figure 1. To prevent data leakage, the dataset was split into three sets: training (70%), validation (20%), and testing (10%). The training subset was only augmented with horizontal and vertical flipping.

### 2.2. SAVE (Spectrum-Aided Visual Enhancer)

In this research, the visual enchantment mechanism called SAVE is employed to convert the RGB dermoscopy images to HSI and to use the BS technique to obtain the necessary band that helps to enhance the visible quality of the cancer lesion. At the first stage of the conversion process, the correlation between the RGB image and the spectrometer was established for multiple RGB values. For the color calibration process, a Macbeth color checker is used. This tool consists of 24 squares showcasing a range of color samples commonly found in nature, including shades like red, green, blue, cyan, magenta, and yellow, as well as six tones of gray. The images are captured and stored based on RGB values; the values are converted to sRGB values in the range between 0 and 1 by dividing by 225. After this process, a gamma correction function is used to transform sRGB values into linearized RGB values. Once the values are linearized to RGB, they are translated to the CIE 1931XYZ color space using the translation matrix. This CIE 1931 color space gives the relation between the visible spectrum and the color experienced in nature. On the camera side, some problems are faced, like a non-linear response, dark current, bad color separation, or color distortion while capturing the image. The process of retrieving the correction matrix is shown in Equation (1). Equation (2) shows the formula for getting the corrected XYZ value.

(1) $$\left[C\right]=\left[{XYZ}_{Spectrum}\right]\times pinv\left(\left[V\right]\right)$$

(2) $$\left[{XYZ}_{Corrcnt}\right]=\left[C\right]\times \left[V\right]$$

On the other hand, spectrometer reflection spectral data is collected and converted into XYZ color space values shown in Equations (3)–(6).

(3) $$X=k\int_{400nm}^{700nm}S\left(\lambda \right)R\left(\lambda \right)\bar{x}\left(\lambda \right)d\lambda$$

(4) $$Y=k\int_{400nm}^{700nm}S\left(\lambda \right)R\left(\lambda \right)\bar{y}\left(\lambda \right)d\lambda$$

(5) $$Z=k\int_{400nm}^{700nm}S\left(\lambda \right)R\left(\lambda \right)\bar{z}\left(\lambda \right)d\lambda$$

(6) $$k=100/\int_{400nm}^{700nm}S\left(\lambda \right)\bar{y}\left(\lambda \right)d\lambda$$

The reflectance spectrum of 24 color patches was then identified with the Ocean Optics QE65000 spectrometer (Ocean Optics, Dunedin, FL, USA), which works with an X-Rite board. The brightness ratio was obtained from the Y value in the XYZ color gamut space since it directly correlated with perceived brightness. The reflectance spectrum data were transformed into XYZ values and normalized. To estimate the correction coefficient matrix (m), multiple regression was employed utilizing Equation (7). The reflectance spectrum data were also applied to identify the transformation matrix (M) for the colors on the X-Rite board. Further, analysis through a principal component analysis (PCA) technique revealed that six principal components and their eigenvectors explained 99.64% of the total variability that exists in the reflectance spectrum dataset. The root mean square error (RMSE) value of the corrected XYZ values when fitted with the spectrum data for the 24 colors was established to be 0.19.

The RGB-to-XYZ transformation allows for the conversion of traditional RGB data to a device-independent color representation. This step is the basis for further spectral reconstruction steps in the SAVE framework and for estimating spectral reflectance information from an RGB dermoscopic image.

Particularly, when selecting the variable $V_{color}$, much attention was paid, as this variable unites all the mentioned combinations of the values of the X, Y, and Z variables.

(7) $$\left[M\right]=\left[Score\right]\times pinv(\left[V_{Color}\right])$$

(8) $${\left[S_{Spectrum}\right]}_{380\sim 780nm}=\left[EV\right]\left[M\right][V_{Color}]$$

Equation (8) shows the way the analog spectrum ($S_{Spectrum}$) was calculated. Subsequently, the performance of $S_{Spectrum}$ was evaluated against the reflectance spectrum ($R_{Spectrum}$). The mean RMSE arrived at 0.056 for 24 color blocks when comparing the $S_{Spectrum}$ and $R_{Spectrum}$, and the average color difference between the acquisition $S_{Spectrum}$ and the $R_{Spectrum}$ produced by the spectrometer was only 0.75. These results clearly explain that it is possible to convert the RGB image into an HSI image.

After converting the RGB images to HSI spectral data, the HSI conversion mechanism generates a large amount of spectral information across various wavelengths. Identifying the optimal spectral signature for an object detection model from this spectral data can be highly time-consuming. To eliminate this time-consuming process, this study focuses on directly utilizing the spectral data generated by Olympus endoscopy by maintaining a similar spectral profile to the narrow-band (NBI) images produced by Olympus endoscopy. The band selection process begins with color calibration, employing a Macbeth color checker. By comparing the generated HSI images against the Olympus spectroscopy NBI images, the CIDE 2000 color difference between the SAVE image and the actual NBI image is calculated and minimized. After all corrections, the color difference between the simulated image and the Olympus endoscopy image is only 2.97, which is negligible. The key factors influencing the color difference include the light spectrum’s color-matching function and the reflection spectrum. There is a significant CIDE 2000 color difference between Olympus endoscopy RGB images and those images captured by digital cameras. This discrepancy arises from differences in the light spectrum used in both systems. However, the intensity will be similar at certain wavelengths; a substantial difference exists in the wavelength 450–540 nm range, and wavelengths in this range are highly absorbed by hemoglobin. The calibration of the light spectrum is the vital point, and it is done using the Cauchy–Lorentz visiting distribution, as shown in Equation (9).

(9) $$f\left(x;x_{0,}\gamma \right)=\frac{1}{\pi \gamma \left[1+{\left(\frac{x-x_{0}}{\gamma }\right)}^{2}\right]}=\frac{1}{\pi }\left[\frac{\gamma }{{\left(x-x_{0}\right)}^{2}+\gamma^{2}}\right]$$

**Figure 2:**
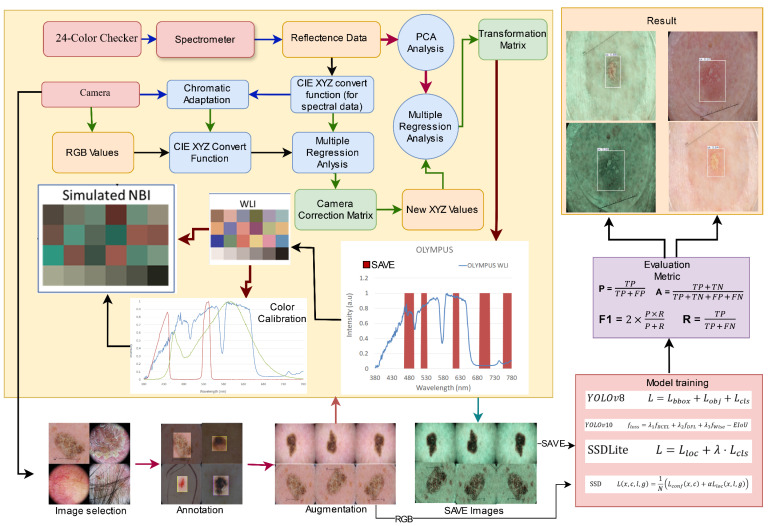
Systematic workflow of skin cancer detection. The color calibration plot shows the spectral responses used during calibration, where the red, green, and blue curves represent the reconstructed spectral channel responses, and the blue line represents the Olympus WLI reference spectrum.

Then, a 24-color checker is employed to further calibrate the SAVE image by comparing it with the Olympus NBI image. The light spectrum is optimized by utilizing the annealing optimization method, resulting in an average standard CIDE 2000 color difference of 5.36, which is also negligible. Real NBI images from the Olympus endoscope include not only green and blue wavelengths but also various shades of brown corresponding to 650 nm. This indicates the use of advanced image post-processing techniques that enhance the realism of NBI images. To achieve realistic outputs, like the Olympus NBI, unspecified regions in Olympus, like 600 nm, 700 nm, and 780 nm, along with specified regions at 415 nm and 540 nm, are used to create the SAVE images. Figure 2 shows the overall schematics of the research.

Band selection refers to the regions of the spectrum that are selected for the diagnostic information they contain and the fact that they do not carry a lot of redundant spectral information. This process helps the SAVE framework focus on the lesion’s features and accentuates the difference between the healthy and abnormal skin tissue.

The conventional methods for spectral reconstruction are all developed to provide accurate hyperspectral reflectance information from RGB inputs for spectral fidelity analysis. The proposed SAVE framework is tailored to medical image enhancement and detection of skin lesions, in contrast. SAVE does not try to acquire the entire hyperspectral cube for physical spectral analysis but selectively aims for and enhances diagnostically relevant spectral features based on principles of band selection (narrow-band imaging). The targeted enhancement method enhances the visibility of lesions and improves the object detection performance without the complexity and the computational requirements of the whole hyperspectral acquisition systems.

The mathematical formulations have been incorporated in this work to describe the proposed SAVE framework in a reproducible and scientifically sound way and to avoid opaqueness in the reconstruction and enhancement of the spectrum.

The XYZ tristimulus values computed before and after color calibration of the 24 color checker reference patches used in the evaluation of spectral reconstruction are shown in Table 2. The root mean square error (RMSE) and standard deviation (SD) are reported to document the accuracy, consistency, and stability of the calibration procedure. Low average RMSE (0.19) and standard deviation (0.39) show that the proposed calibration procedure has good color correction and spectral reconstruction performance, which ensures that the SAVE framework is robust.

### 2.3. Intuitive Overview of the SAVE Framework

The SAVE framework can be seen from a clinical point of view as a spectral enhancement pipeline aimed at increasing the visibility of lesions with normal RGB dermoscopic images. SAVE estimates diagnostically relevant spectral information from RGB data without the need for specialized hyperspectral imaging hardware, identifies informative wavelength regions, and produces enhanced images that can be used to enhance lesion detection using deep learning models. The goal is to maintain clinically relevant characteristics of the lesions and to maximize visual contrast and spectral differentiation.

### 2.4. Implementation Details and Reproducibility

All dermoscopic images were resized to a size of 640 × 640 pixels before processing. The SAVE pre-processing pipeline was programmed in Python 3.10, with the help of OpenCV 4.12, NumPy 2.3, and Scikit 1.7.1-learn libraries. RGB images were first normalized to sRGB in the range [0–1] and then gamma-corrected and transformed to CIE 1931 XYZ color space.

The Macbeth color checker, which is a standard set of color patches, was used to perform spectral calibration and estimation of the correction matrix. The spectral redundancy was reduced using principal component analysis (PCA) with six principal components that explain 99.64% of the spectral variance used for reconstruction.

The spectral enhancement stage employed the spectral regions 415 nm (centered at 415 nm), 540 nm (centered at 540 nm), 600 nm (centered at 600 nm), 700 nm (centered at 700 nm), and 780 nm (centered at 780 nm) as narrow band imaging modes to simulate the narrow band imaging characteristics of the Olympus endoscopic imaging systems. CIEDE2000 color difference between reconstructed SAVE images and reference narrow-band images has been minimized by simulated annealing optimization.

They were trained using a Kaggle NVIDIA Tesla P100 GPU environment for 500 epochs, with a batch size of 32 and a confidence threshold of 0.60. The default hyperparameters were kept as provided in the PyTorch 2.6 implementations, unless otherwise stated.

### 2.5. Computational Complexity Analysis

The proposed SAVE framework is composed of several pre-processing stages such as RGB to XYZ color transformation, spectral reconstruction, principal component analysis (PCA), band selection, and image enhancement. Many are used on a per-pixel basis and thus have approximately linear computational complexity with respect to the image size. To combat spectral redundancy and to reduce the dimensionality of the reconstructed spectral information to lower the computational needs in subsequent steps, PCA is used.

The SAVE framework adds some pre-processing time above the conventional RGB-based workflows, but it is still efficient in practice. The experimental results demonstrated that the entire pre-processing pipeline for the SAVE framework was able to reach about 10.6 frames per second (FPS) on a standard GPU-based system. The computational complexity of the following object detection models does not change because SAVE is done prior to deep learning inference. These results show that the proposed method is computationally efficient enough to be practically applied to computer-aided dermatological image analysis and processing large datasets in the field.

### 2.6. ML Algorithms

#### 2.6.1. YOLOv8

YOLOv8 is an updated version of the deep learning model YOLOv5, developed specifically in the field of object detection. It includes multiple network structures and uses C2F modules to improve YOVOv5 [39]. YOLOv8 has a new backbone network, which is an optimized version of CSPDarknet53 that comprises 53 convolution layers in total; the system applied cross-stage partial connections to efficiently transfer information through the different levels of the network [40]. It is built primarily based on the deep learning framework PyTorch, and the key mechanisms are the C2F module, efficientrep, distributed focal loss (DFL), and complete intersection over union (CIoU) [41]. Overall loss of the prediction is simply obtained by the sum of bounding box loss, objectness loss, and classification loss, and it is mentioned in Equation (11).

(10) $$L=L_{bbox}+L_{obj}+L_{cls}$$

#### 2.6.2. YOLOv10

YOLOv10 was introduced by researchers at Tsinghua University and shown in May 2024 [42]. YOLOv10 is further improved by the parallel split attention (PSA) and compact inverted bottleneck (CIB) blocks, which help to perform multi-scale feature processing and effective attention mechanisms [43]. The consistent matching metric is expressed in Equation (11) [44].

(11) $$m=s\times p^{a}\times IoU{\left(b^{\prime },b\right)}^{\beta }\;\;$$

This overall loss function is derived from binary cross-entropy loss, distribution focal loss, weighted intersection over union, and enhanced intersection over union. It is shown in Equation (12) [45].

(12) $$f_{loss}=\lambda_{1}f_{BCEL}+\lambda_{2}f_{DFL}+\lambda_{3}f_{Wise}-EIoU$$

All these design choices also emphasize the fact that YOLOv10 is designed to be optimized to solve more challenging object detection problems with higher accuracy and efficiency [46].

#### 2.6.3. SSDLite

The SSDLite model used in this study is implemented from the PyTorch object detection models module. It is selected due to its ability to predict fast and accurately [47]. SSDLite uses Type 3 prior boxes for the first scale feature maps and Type 2 prior boxes for the other feature maps [48,49]. The architecture of mobilenetV3 is composed of a series of bottleneck blocks. Some of the bottleneck blocks include residual structure [50]. For higher accuracy, a new kind of activation function called swish is introduced to replace the rectified linear unit (ReLU) function, as shown in Equation (13).

(13) $$s\;swish\left(x\right)=x^{\circ }\sigma \left(x\right)\;\;$$

This results in the hard version of swish (h-swish), and it is shown in Equation (14) [51].

(14) $$h\;swish\left[x\right]=x\frac{ReLU6\left(x+3\right)}{6}$$

And overall loss is derived from three values: localization loss, classification loss, and regularization loss. The equation for finding overall loss is shown in Equation (15) [52]:

(15) $$L_{total}=L_{loc}+L_{cls}+\lambda \sum_{i=0}^{n}\omega_{i}^{2}$$

#### 2.6.4. SSD

In the current work, a single-shot detector (SSD) model in PyTorch with a VGG16 base network is also trained. The SSD300 approach is a feed-forward convolutional network with the output of a fixed-size set of bounding boxes and the corresponding scores stating the probability of the objects of a certain class present in those boxes. A non-maximum suppression step is then ensured to fine-tune the end detection results further [53]. The SSD training objective is inspired by the multi-box objective but is further generalized for multiple object classes. The overall objective loss function is a weighted sum of the localization loss and the confidence loss. It can be expressed in Equation (16):

(16) $$L\left(x,c,l,g\right)=\frac{1}{N}\left(L_{conf}\left(x,c\right)+\alpha L_{loc}(x,l,g)\right)\;\;$$

Here, N is the number of default boxes that match the ground truth, and for localization loss, L1 smooth loss between the predicted box and the ground box is used. To improve the performance of the model, the tiling of the default boxes is decided according to the feature maps that respond to specific object scales. If m feature maps are used for prediction, the scale of the default boxes for each feature map is shown in Equation (17) [54]:

(17) $$s_{k}=s_{min}+\frac{s_{max}-s_{min}}{m-1}\left(k-1\right),\;\;k\in \left[1,m\right]\;\;$$

YOLOv11: YOLOv11 is the newest version in the YOLO family, and it has an improved backbone and neck structure. This upgrade enhances its feature extraction tasks, leading to better object recognition and, more importantly, an increased ability to deal with complex tasks. YOLOv11 has brought major improvements in architectural components and optimized training mechanisms for faster processing, but it still maintains the best degree of accuracy. These improvements enable it to obtain increased mean average precision with fewer parameters [55].

## 3. Results

The following section compares the performance of deep learning-based object detection models—YOLOv8, YOLOv10, YOLOv11, SSDLite, and SSD—in detecting AK, BCC, and SK skin cancer lesions across both RGB and SAVE datasets. As detailed in Table 3 below, the YOLOv8 model demonstrates an approximate 5% increase in accuracy when utilizing the SAVE dataset compared to the standard RGB dataset. The findings reveal that the SAVE mechanism improves precision to 100% for BCC while maintaining the same recall level as the RGB approach. This clearly indicates that SAVE uncovers previously hidden information not captured in conventional RGB images. Similarly, for SK detection, the model exhibits a high recall on the SAVE dataset—an increase of approximately 10% over the RGB-based model. Because early-stage SK closely resembles unaffected skin, the RGB-based model may overlook crucial details; however, the SAVE-based model shows notable improvements due to its ability to extract additional spectral data. This previously concealed information significantly enhances the performance of YOLOv8.

Furthermore, a comparison of the datasets reveals that YOLOv10 performs significantly better on the SAVE dataset compared to its RGB counterpart and the YOLOv8 model. This improvement is achieved by the optimized backbone and neck design of YOLOv10, which fully exploits the unique hidden information within the SAVE dataset, allowing it to earn 100% precision for AK. The results also highlight the performance of SSDLite, which achieves about 90% accuracy on the SAVE dataset, representing roughly a 4% improvement over its RGB performance. Impressively, the model’s recall for BCC reached nearly 100% on the SAVE dataset. This indicates that in a single forward pass, the model can learn vital features that are not discernible in standard RGB images.

Moreover, the upcoming table illustrates a drastic improvement in the SAVE-based SSD model compared to all other evaluated architectures. Due to its single forward pass capability, this model effectively and quickly extracts information without overfitting. On the SAVE dataset, the SSD model achieves 100% precision for both AK and BCC detection, alongside 100% recall for SK detection. These results reveal that for skin cancer detection, the SSD model utilizing the SAVE dataset yields optimal performance. Finally, the YOLOv11 results clearly demonstrate recent advancements in object detection. Showing a gradual improvement over earlier iterations, YOLOv11 maintains strong performance on the SAVE dataset with no significant drop compared to the RGB-based model. Although there is a minor decrease in BCC recall (less than 3%), the precision simultaneously increased by more than 3%, and all other evaluation metrics show noticeable improvements.

**Figure 3:**
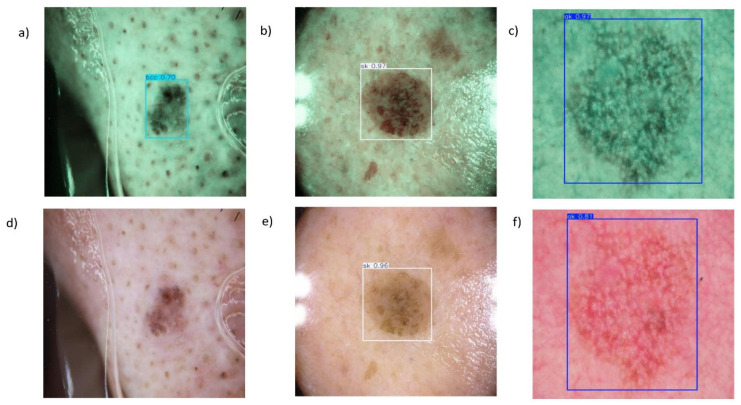
Resulting images predicted from the RGB dataset: (**a**) BCC SAVE; (**b**) SK SAVE; (**c**) AK SAVE; (**d**) BCC RGB; (**e**) SK RGB; (**f**) AK RGB.

In Figure 3, images (a), (b), and (c) represent the SAVE images of BCC, SK, and AK. Similarly, (d), (e), and (f) represent the RGB images of BCC, SK, and SK images. There is a noticeable improvement in SAVE images compared to RGB images in a general view. For instance, to compare images (a) and (d), both present the same BCC image; however, there was no detection in the RGB image. The RGB image lacked sufficient detail for the model to identify the affected skin lesions, as there was minimal difference between the affected and normal skin. In contrast, the SAVE mechanism enhances the visual differentiation between the affected and normal skin lesions, allowing the deep learning model to successfully detect the affected areas in the SAVE dataset. While comparing images (b) and (e), both figures seem mostly identical at first glance, but the SAVE mechanism makes the affected areas stand out much more. The RGB image is faint when comparing normal skin areas to affected areas, while in the SAVE dataset, normal and affected skin lesion areas are demarcated very well. This differentiation is critical for the correct positioning of features of localization and bounding boxes around the affected regions. It is mainly done by removing the unwanted spectral information from the images. That way, the remaining colors interfere less with the object detection algorithm due to the removal of dominant colors. The comparison between images (c) and (f) is vivid evidence of the enhancements provided through the SAVE mechanism. In this case, one of the proposed models, YOLOv10, trained with the SAVE dataset, has a 15% confidence boost as per the experiment results compared to the detection confidence of the YOLOv10 model trained with the RGB dataset. This happened because of minimal visual efficiency in RGB images; there is no difference between affected and normal skin lesions. On the other hand, the SAVE mechanism improves the contrast between healthy skin and affected skin. Often, the affected skin lesions in early-stage skin cancers look almost clinically identical to normal skin, but a trained dermatologist can identify slight differences, which may be difficult for a deep-learning model based on conventional RGB to discern. However, the visual enhancement techniques used in SAVE make it possible for the algorithm to function substantially better in such cases. The overall analysis reveals that all the models achieve a higher level of accuracy when trained with SAVE rather than the RGB dataset. For the SAVE dataset, the SSD model outperforms the other models, achieving the highest accuracy of 96%, a total precision of 97%, and a recall of 96%.

The SAVE-based models showed consistently better performance than the RGB-based models, but these results should be viewed with some degree of caution owing to the relatively small dataset and the lack of an independent external test set. Even so, statistical analyses revealed that SAVE enhancement led to statistically significant improvements in multiple evaluation measures.

The selected wavelength regions were chosen based on biomedical relevance and optical interactions with skin tissue. The wavelength of around 415 nm is a peak of hemoglobin absorption and is the best wavelength to emphasize superficial vascular structures and microvascular patterns. Similarly, the 540 nm region is related to hemoglobin absorption and leads to better visualization of vascular structures and lesion-related vascular properties. The information at the difference wavelength of 600 nm is complementary to tissue pigmentation, lesion morphology, and chromophore distribution. At longer wavelengths of 700 nm and 780 nm, the depth of tissue penetration is greater and has the ability to capture structural information from deeper skin layers. The proposed SAVE framework can boost information diagnostically relevant to wavelength regions and facilitate the visual separation of pigmented lesion tissue from healthy skin without compromising the compatibility with conventional RGB dermoscopic imaging. Figure 3 illustrates representative lesion detection results generated by the evaluated deep learning models using both RGB and SAVE-enhanced images.

## 4. Discussion

This study explains the performance improvements of deep learning models while using the SAVE mechanism applied to dermoscopy images over normal RGB images. The outstanding results of the deep learning models explicitly show that the SAVE mechanism works perfectly for enhancing the viewing quality of the affected skin lesions and showcasing the hidden details in the images. Though the deep learning model has a high level of accuracy, it also has some shortcomings. As the largest organ of the human body, the skin is affected frequently by different conditions, but in this study, we only utilized the three most frequent skin conditions. Due to its minimal range of class knowledge, the model misclassifies some common skin conditions, such as skin allergy, heat burn, and damaged pimples, as critical skin cancers mentioned in this research. However, we chose data from a large dataset, like ISIC 2019, and the images were collected from all over the world. Due to some uncompromised conditions, the images selected from the dataset are too minimal compared to a wide range of skin lesion conditions. This led the deep learning model to struggle with inexperienced data from different environments. Even though three frequent skin cancers were worked on in this research, every skin cancer utilized in this research has some stages, but this study focuses on the cancer type instead of its stages. However, we got positive results by directly applying the spectral information of Olympus endoscopy without any previous work reference in skin cancer. There is a high number of possibilities to obtain good results from some other combination of spectral bands or wavelengths. This study mainly focuses on the output of five trained models, although various object detection models with a variety of learning approaches were available. Another limitation of the SAVE mechanism is that the generated extra information sometimes may lead to some unwanted decisions or overfitting problems in high-weightage models, like Faster RCNN, with a ResNet backbone. The other negligible limitation of the SAVE mechanism, when it is applied to RGB images, is that the deep learning models take more training time to fine-tune the model due to the additional data produced by the SAVE mechanism.

The future scope of this study includes several promising avenues for improvement. Incorporating a wider variety of skin conditions would enhance the model’s generalization capabilities, while investigating different stages of these conditions could improve classification accuracy. Additionally, utilizing transfer learning techniques could boost performance, especially with limited datasets. Testing the model across diverse populations will ensure broader applicability. The next significant thing to improve the effectiveness of the object detection model is to combine the efficiency of multiple models. This approach can be exploited to improve the general object detection accuracy by a high percentage. Finally, developing user-friendly tools for clinical use will facilitate adoption by healthcare professionals.

Although the SAVE-based models achieved high precision and recall values under the current experimental conditions, these results should be interpreted cautiously. The relatively limited dataset size, restricted lesion diversity, and absence of independent external validation may contribute to optimistic performance estimates. While the findings demonstrate the potential of the proposed SAVE framework, further evaluation using larger and more diverse datasets, multi-center validation studies, and prospective clinical assessment is required before broader clinical applicability can be established.

One interesting fact that can be seen from the experimental results is that the SSD architecture performed better than the recent YOLO architecture-based ones. The advanced optimization strategies and richer feature extraction mechanisms in YOLOv8, YOLOv10, and YOLOv11 usually come with larger and more diversified datasets. By contrast, the limited number of images in this study could have benefited the simpler SSD architecture by minimizing the risk of overfitting and the complexity of the model. Moreover, SSD detects multiple levels of feature maps with different sizes, which can be beneficial for detecting the lesion structures and boundaries enhanced by the SAVE pre-processing framework. The results indicate that the suitability of the models might be related to dataset characteristics, pre-processing methods, and architectural sophistication.

In order to maintain the morphology and spectral features of the lesion, conservative augmentation methods were used, but future studies will investigate other augmentation methods such as brightness variation, contrast adjustment, rotation, scaling, and geometric transformation for further development of the robustness and generalization of the models across a variety of imaging conditions.

### 4.1. Examples of Failure Cases

As shown in Table 4, the proposed framework has been shown to have some difficulties for certain representative examples of lesions with low contrast, severe illumination variation, and irregular texture patterns, or lesions that are visually similar to benign dermatological conditions. In some instances, the skin artifacts (occlusion by the hair or pigmentation around the skin) led to wrong localization and/or lower detection confidence.

These results indicate that SAVE can enhance the contrast but is still sensitive to the variability of image quality and lesion heterogeneity. Future research will concentrate on the introduction of bigger and more varied datasets, stiffening pre-processing methods to resist artefacts, and explainable AI techniques to boost the reliability of the approach in real-world clinical contexts.

The relatively small dataset size could lead to overfitting and a lack of generalizability to different clinical settings. To overcome this effect, the train/validation/test separation was carefully carried out prior to augmentation, and augmentation methods were limited to transformations that are realistic (e.g., horizontal and vertical flipping). However, further studies will involve more large-scale multi-center datasets and external validation to assess the robustness of the proposed SAVE framework further.

The SAVE framework showed potential in performance, but the reconstruction of the spectra still relies on the quality of the calibration and the strategies to select the wavelength. Further work will involve the study of adaptive spectral optimization and fully automated reconstruction pipelines for further enhancement of the robustness and repeatability of the reconstruction.

While the proposed SAVE framework showed encouraging improvement in the tasks of skin lesion detection, the present study is limited to evaluating the proposed framework on the ISIC2019 dataset, which was filtered and used in a retrospective manner. Further validation of external data, collected in independent multi-center studies, is still needed before clinical use can be advocated.

Further, there is no direct comparison of the dermatologists’ performance nor a prospective assessment of the clinical workflow in the present study. Hence, the SAVE framework is presently more of a computer-assisted diagnostic support mechanism and not a clinical decision-making mechanism.

However, this proposed enhancement mechanism could be useful in resource-constrained environments since it does not need any special hyperspectral acquisition hardware. The improvement of the visibility of the lesions in the standard dermoscopic images, with the use of SAVE, could help the clinician during the preliminary lesion screening and diagnostic interpretation.

As noted, the high precision and recall results seen in some experiments can be at least partially attributed to the fact that the conditions of the datasets were relatively controlled and there was a limited number of lesions studied. To minimize the risk of overfitting, augmentation and train–validation–test separation techniques were used to create a more compact framework; additional evaluation with larger and more diverse datasets is necessary to validate the robustness and generalizability of the proposed framework.

### 4.2. Limitations

It was not the aim of the present study to make a direct comparison with performance by dermatologists. Further research will consist of reader studies with dermatologists and prospective clinical validation to assess the potential of SAVE to improve clinical use.

Variations in dermoscopic devices, illumination conditions, skin tone diversity, and lesion acquisition protocol can have an impact on the real-world deployment. Thus, more validation in diverse clinical contexts is warranted for robustness and generalizability.

The proposed SAVE framework showed promising improvement in the performance of lesion detection in AK, BCC, and SK, but the present study did not truly reflect the diversity of dermatologic disease seen in real-world clinical practice. The present structure was particularly developed to assess lesions with imperceptible visual characteristics, and it was difficult to diagnose low-contrast features in simple RGB dermoscopy. Thus, for a preliminary evaluation of the SAVE-based spectral enhancement mechanism, the three lesion categories of actinic keratosis (AK), basal cell carcinoma (BCC), and seborrheic keratosis (SK) were chosen.

In clinical practice, however, dermatological sessions are far more complex with the presence of melanoma, benign nevi, inflammatory skin diseases, vascular abnormalities, allergic reactions, pigmented lesions, and other benign dermatological diseases that can have similar visual features. This lack of these extra categories of lesions might reduce the clinical generalizability and robustness of the current framework in more general clinical settings.

In addition, the small number of cases and limited number of lesions could complicate the possibility of overfitting and decrease the generalization ability to other clinical situations, which are often highly heterogeneous. More extensive validation with larger and more diverse datasets is needed, but careful separation of the train/validate/test sets and conservative augmentation of the datasets were used to minimize possible bias and to avoid the loss of any diagnostic lesion morphology.

Future studies will aim to make the proposed SAVE framework more clinically applicable and robust by adding more dermatologic conditions such as melanoma, benign nevi, inflammatory skin diseases, and vascular disease. Other studies with larger multi-class datasets and external multi-center validation will follow to further assess the generalizability and reliability of the framework in various clinical settings. Advanced augmentation techniques, transfer learning approaches, ensemble learning techniques, and adaptive spectral optimization techniques will also be explored in future research to further enhance the robustness of the model as well as the detection performance. In addition, explainability techniques will be adopted to improve the interpretability and visualization of the attention regions of lesions of clinical interest (Grad-CAM and SHAP). Lastly, the potential of translating the SAVE framework for dermatologic screening applications in real-world settings will be explored by evaluating the potential workflow for clinical workflow evaluation and comparing clinical performance with that of dermatologists.

## 5. Conclusions

In conclusion, skin cancer remains a significant public health problem, particularly among older people. The increasing incidence of NMSC highlights the importance of early detection and treatment. This study proves that the SAVE method is more accurate than the conventional RGB-based approach in recognizing skin cancer lesions such as AK, BCC, and SK. The experimental results show that significant improvements can be observed in each deep learning model when trained with the SAVE dataset. Although all models showed improvement, the SSD model stood out with exceptional performance metrics. The evaluation of the model shows a 96% accuracy rate, a 96% recall rate, 97% precision, and a 96% F1 measure. By contrast, the RGB-based model maintained a 95% accuracy, recall, precision, and F1 score. The above outcomes prove that using SAVE outperforms traditional RGB imaging for the identification of skin cancer lesions.

## Figures and Tables

**Table 1:** Dataset before and after filtering.

Class	Before Filtering	After Filtering
AK	867	257
BCC	3323	286
SK	1316	322
Total	5506	865

**Table 2:** RMSEs of the XYZ values before and after calibration.

S.No	Before Calibration	Before Calibration	Before Calibration	After Calibration	After Calibration	After Calibration	RMSE	SD
	X	Y	Z	X	Y	Z		
1	10.96	9.92	4.63	11.14	9.87	4.26	0.24	0.30
2	38.74	35.80	18.65	38.57	35.94	18.66	0.13	0.08
3	16.62	19.07	24.13	16.48	18.79	24.11	0.18	0.17
4	10.33	12.86	4.62	10.16	13.03	4.85	0.19	0.19
5	24.05	23.87	31.55	24.16	24.07	31.60	0.13	0.08
6	30.12	42.15	32.40	30.10	42.17	32.42	0.02	0.002
7	38.10	30.24	4.28	38.04	30.37	4.22	0.09	0.04
8	11.70	11.47	25.90	11.64	11.37	25.91	0.07	0.02
9	29.01	19.91	9.62	29.20	19.78	9.60	0.13	0.08
10	8.26	6.49	9.63	8.06	6.49	9.86	0.18	0.17
11	34.15	44.06	8.44	34.15	44.02	8.53	0.06	0.01
12	47.99	44.55	6.05	48.05	44.34	6.17	0.15	0.11
13	6.82	5.79	21.07	6.90	5.91	21.00	0.09	0.04
14	14.55	23.55	7.22	14.58	23.51	7.12	0.07	0.02
15	21.08	12.25	3.57	21.01	12.28	3.65	0.06	0.01
16	58.40	60.69	7.54	58.38	60.79	7.42	0.09	0.04
17	28.98	19.54	20.67	28.94	19.52	20.66	0.02	0.002
18	12.81	19.01	28.54	12.84	19.10	28.56	0.05	0.01
19	82.12	88.54	67.20	82.31	88.73	67.51	0.24	0.30
20	54.74	58.92	45.52	54.28	58.40	44.75	0.60	1.89
21	33.08	35.73	27.24	33.26	35.82	27.54	0.21	0.23
22	18.18	19.62	14.94	18.86	20.31	15.62	0.68	2.43
23	9.13	10.01	8.13	8.56	9.26	7.21	0.76	3.04
24	2.87	3.19	2.39	3.10	3.35	2.68	0.23	0.27
**Average**							**0.19**	**0.39**

**Table 3:** Performance comparison between detection models on both the RGB and SAVE imaging techniques.

Framework	Model	Classes	Metrics			
			Precision in %	Recall in %	F1 Score in %	Accuracy
YOLOv8	RGB	AK	91.1	70.6	79.6	77
		BCC	92	79.3	85.2	
		SK	83.9	81.2	82.5	
	SAVE	AK	91.7	75.9	83.1	82
		BCC	100	79.3	88.5	
		SK	90.9	93.8	92.3	
YOLOv10	RGB	AK	95.5	72.4	82.3	78
		BCC	91.7	75.9	83.1	
		SK	90	84.4	87.1	
	SAVE	AK	100	79.3	88.5	84
		BCC	92	79.3	85.1	
		SK	96.7	90.6	93.5	
SSDLite	RGB	AK	79.17	82.61	80.85	86
		BCC	89.29	92.59	90.91	
		SK	88.89	82.76	85.71	
	SAVE	AK	90.91	80	85.11	90
		BCC	86.67	100	92.86	
		SK	93.1	90	91.53	
SSD	RGB	AK	95.83	92	93.88	95
		BCC	93.1	100	96.43	
		SK	96.43	93.1	94.74	
	SAVE	AK	100	91.67	95.65	96
		BCC	100	96.3	98.11	
		SK	90.91	100	95.24	
YOLOv11	RGB	AK	91.7	75.9	83.2	81
		BCC	93.1	92.6	92.8	
		SK	89.7	81.2	85.3	
	SAVE	AK	96.8	82.8	89.3	84
		BCC	96.3	89.7	92.9	
		SK	96.4	84.4	90	

**Table 4:** Representative examples of failure cases observed in the proposed SAVE-based skin lesion detection framework.

Problem	Example
false positive	benign skin texture is classified as AK
false negative	low-contrast BCC is missed
confusion	SK is confused with an SCC-like lesion
artifact issue	hair occlusion causing wrong detection

## Data Availability

The data presented in this study are available upon request from the corresponding author. The data are not publicly available due to strict privacy and ethical restrictions mandated by the Institutional Review Board of Ditmanson Medical Foundation Chia-Yi Christian Hospital (Approval No. IRB 2025059).
